# Inflammatory Mediator Profiling Reveals Immune Properties of Chemotactic Gradients and Macrophage Mediator Production Inhibition during Thioglycollate Elicited Peritoneal Inflammation

**DOI:** 10.1155/2013/931562

**Published:** 2013-03-31

**Authors:** Derek Lam, Devon Harris, Zhenyu Qin

**Affiliations:** Division of Vascular Surgery, Department of Surgery, University of Texas Health Science Center at San Antonio, San Antonio, TX 78229, USA

## Abstract

Understanding of spatiotemporal profiling of inflammatory mediators and their associations with MΦ accumulation is crucial to elucidate the complex immune properties. Here, we used murine thioglycollate elicited peritonitis to determine concentrations of 23 inflammatory mediators in peritoneal exudates and plasma before (day 0) and after (days 1 and 3) thioglycollate administration to peritoneal cavities; these mediators included TNF-**α**, FGF-9, IFN-**γ**, IP-10, RANTES, IL-1**α**, IL-6, IL-7, IL-10, IL-11, IL-12p70, IL-17A, lymphotactin, OSM, KC/GRO, SCF, MIP-1**β**, MIP-2, TIMP-1, VEGF-A, MCP-1, MCP-3, and MCP-5. Our results showed that concentrations of most mediators in exudates and plasma reached peak levels on day 1 and were significantly reduced on day 3. Conversely, MΦ numbers started to increase on day 1 and reached peak levels on day 3. Moreover, LPS treatment *in vitro* significantly induced mediator productions in cell culture media and lysates from MΦ isolated on day 3. Our results also showed that on day 0, concentrations of many mediators in plasma were higher than those in exudates, whereas on day 1, the trend was reversed. Overall, the findings from thioglycollate elicited peritonitis reveal that reversible chemotactic gradients between peritoneal exudates and blood exist in basal and inflamed conditions and the inflammatory mediator production *in vivo* is disassociated with macrophage accumulation during inflammation resolution.

## 1. Introduction

During the early stage of inflammatory diseases, the gradients of some of the inflammatory mediators play important roles in the recruitment of leukocytes to inflamed areas. In this stage, monocytes and macrophages (MΦ) are the secondary line of inflammatory cells after neutrophils. In contrast to the recruited neutrophils with short life spans due to apoptosis [1], MΦ have longer life spans and play a more important role in the clearance of neutrophils via phagocytosis. In addition to leukocyte recruitment, another primary response to inflammation is the secretion of the inflammatory mediators, including both proinflammatory mediators such as interleukin (IL)-1, IL-6, and tumor necrosis factor (TNF)-*α*, and anti-inflammatory mediators such as IL-10. These mediators are produced mainly by leukocytes within the inflamed areas and they elicit particular immune responses to target cells or tissues via specific receptors and signaling pathways. Thus, it is important to understand the spatiotemporal association between the inflammatory mediator profiling and leukocyte accumulation. 

Murine thioglycollate elicited peritonitis is an appropriate model with which to study inflammatory events that are accompanied by inflammatory mediator production and leukocyte accumulation. In this model, neutrophil numbers start to increase and reach peak levels between 4 and 24 hours after treatment, while MΦ numbers start to increase at 24 hours and reach peak levels at 3 to 4 days. We reported that copper is accumulated in the peritoneal MΦ lack of ATP7A [2]. The peritoneal cavity is colonized with leukocytes. Under steady conditions, the resident peritoneal cells include MΦ, T cells, B cells, NK cells, mast cells, and dendritic cells [3, 4]. Resident MΦ and dendritic cells provide a basal immune surveillance. Notably, a newly described population in adipose tissue, associated lymphoid clusters, has been observed in the murine peritoneal cavity [5]. In addition to these immune cells, mesothelial cells that line the peritoneum also serve as a source of inflammatory mediators within the peritoneal cavity [6, 7]. Although controversy exists on the subject, the role of resident peritoneal MΦ in thioglycollate elicited murine peritonitis appears to be minimal. For example, following the selective depletion of resident MΦ (86% reduction) by pretreatment of mice with liposomes containing Cl_2_MDP (clodronate), thioglycollate administration did not alter neutrophil accumulation into peritoneal cavities [8]. A similar finding also occurred when mast cells were selectively depleted (95% reduction) by the pretreatment of mice with compound 48/80 [8]. Interestingly, resident MΦ depletion inhibited neutrophil influx in lipopolysaccharide (LPS) induced peritonitis, whereas MΦ depletion increased neutrophil influx in zymosan induced peritonitis [8]. Previously, studies also showed that thioglycollate treatment ousted nearly all of the resident peritoneal MΦ in the draining lymph nodes within 4 hours [9, 10]. Although large-scale studies have been performed to investigate the association between thioglycollate induced inflammatory mediator production and peritoneal leukocyte accumulation *in vivo*, these studies were usually designed to elucidate elegant mechanisms that were related to the functions of specific proteins in these events. Therefore, a global view is needed of the inflammatory mediator profiling and its association with leukocyte accumulation. Thus, we recently investigated the concentrations of multiple inflammatory mediators in peritoneal exudates and plasma before (day 0) and after (day 1 and 3) thioglycollate administration to murine peritoneal cavities. This study reveals important immune properties that are related to chemotactic gradients and the inhibition of MΦ mediator production *in vivo*. 

## 2. Materials and Methods

### 2.1. Mice

C57BL/6 males (The Jackson Laboratory, Bar Harbor, ME, USA) were used for this study at 10–14 weeks old. The animal protocol was approved by Institutional Animal Care and Use Committee of the University of Texas Health Science Center at San Antonio.

### 2.2. Thioglycollate Induced Peritonitis

Peritoneal cavities were lavaged with 3% Brewer thioglycollate broth (Sigma-Aldrich, St. Louis, MO) to establish acute sterile peritonitis. During the peritoneal exudate collection, mice were excluded from further studies if obvious adhesions were observed between the peritoneum and skin and/or if marked increases were observed in peritoneal thickness of peritoneum. 

### 2.3. Collection of Blood and Peritoneal Exudates

Mice were euthanized at indicated times, and heparinized blood was collected from the hearts. Blood samples were centrifuged at 250 ×g for 5 minutes for plasma isolation. Subsequently, in order to collect peritoneal exudates, one milliliter of 1x phosphate buffered saline (PBS) was administered via intraperitoneal (i.p.) injection. Peritoneal exudates were harvested following 60 seconds of peritoneal massage. After centrifugation at 250 ×g for 5 minutes, the supernatant exudates were collected. Both the plasma and exudate samples were stored at −80°C before further studies. After the first administration of 1 mL of PBS, mice were administered a second dose of 3 mL of 1xPBS. The cell pellets from these two administrations were combined and resuspended in 1 mL of red blood cell lysis buffer (Hybri-Max, Sigma-Aldrich) at room temperature for 1 min. Next, the mixtures were washed with RPMI 1640 medium (Hyclone, Logan, UT) that was supplemented with 10% fetal bovine serum (FBS), 100 units/mL of penicillin, and 100 mg/L of streptomycin. Cells were then pelleted and resuspended in the same growth medium at room temperature for further studies.

### 2.4. Flow Cytometry

Peritoneal exudate cells were first incubated with Fc block (anti-CD16/CD32) (BD Biosciences, San Jose, CA) for 20 min and stained for surface expression using anti-Ly6G-PE (BD Biosciences) and/or anti-F4/80-AF647 (Serotec, Oxford, UK) for 30 min. All washing and staining were performed with 2% FBS in PBS. At least 22,000 cells were analyzed for each sample. The numbers of neutrophils and macrophages were calculated by multiplying the total cell number by the percentage of positive cells for each immunostain. 

### 2.5. Peritoneal MΦ Isolation and Treatment with Lipopolysaccharide (LPS)

Peritoneal exudate cells were placed on polystyrene Petri dishes (95 mm × 15 mm) at 1–0.5 × 10^6^ cells/mL for two hours at 37°C in a humidified 95% air-5% CO_2_ atmosphere. Then these cells were washed twice to remove nonadherent cells. The MΦ purity was confirmed by F4/80 staining and morphology. 

LPS (Sigma-Aldrich) is extracted from *Escherichia coli* serotype O55:B5 and purified by gel filtration chromatography. LPS stock solution was prepared in double distilled water at a concentration of 5 mg/mL. During the experiment, murine peritoneal MΦ at 0.5 × 10^6^ cells/mL were first placed into a 24-well plate with growth area at 2 cm^2^ for 4 h. Cells were then treated 24 h with LPS. After collection of cell culture media, the cell pellets were lysed with 50 mM Tris-HCl with 2 mM EDTA, pH 7.4. Both cell culture media and lysates were stored at −80°C before further studies.

### 2.6. Detection of Inflammatory Mediators

Twenty-three inflammatory mediators were analyzed in plasma, peritoneal exudates, cell culture media, and lysates using a bead-based multiplexing immunoassay (Myriad RBM, Austin, TX) [11, 12]. These mediators included TNF-*α*, fibroblast growth factor (FGF) 9, interferon (IFN) *γ*, IFN-*γ*-inducible protein (IP) 10, RANTES, IL-17A, IL-12p70, IL-11, IL-10, IL-7, IL-6, IL-1*α*, lymphotactin, oncostatin-M (OSM), growth-regulated *α* protein (KC/GRO), stem cell factor (SCF), macrophage inflammatory protein (MIP)-1*β*, MIP-2, tissue inhibitor of metalloproteinase (TIMP)-1, vascular endothelial growth factor (VEGF)-A, monocyte chemotactic protein (MCP)-1, MCP-3, and MCP-5. For the analyses, samples were first thawed, vortexed, and centrifuged before being loaded into a 96-well microtiter plate. Next, the microtiter plate was placed onto a liquid handler machine in which the samples were automatically added to reaction wells that contained capture beads. The beads were incubated with the samples for 1 hour at room temperature to allow the antigens of interest to bind to their targets. Multiplexed cocktails of biotinylated, reporter antibodies for each multiplex were added to the bead mixtures, and were incubated for 1 hour at room temperature. Multiplexes were developed using an excess of streptavidin-phycoerythrin solution that was thoroughly mixed into each multiplex and incubated for 1 hour at room temperature. The plates were washed to remove unbound detection reagents and were read on a Luminex 100 instrument, in which the excitation beams detected the fluorescent signals of each bead. A minimum of 50 beads were detected per protein, per sample. All values were reported as the means. For each multiplex, both calibrators and controls were included on each microtiter plate. This assay was validated by ELISA [13, 14] and has been widely used to determine murine inflammatory mediator profiling at the protein levels [15]. 

### 2.7. Statistics

For each inflammatory mediator, the mean ± standard error (SE) was calculated for each experimental group. Data were compared by Student's *t*-test (two tails). Significant differences were defined as having *P* values < 0.05. For inflammatory mediator detection in plasma and exudates, if more than 50% of the samples from an experimental group were below the detection limit, the group was marked as undetectable. If fewer than (or equal to) 50% of samples from an experimental group were below the detection limit, the least detectable dose was used as the concentration of these samples for further statistics. For the fold increase determination in the LPS treatment studies, if the pretreatment sample values were below the detection limit, the least detectable dose was used to estimate the fold increase. 

## 3. Results

**Figure 1:**
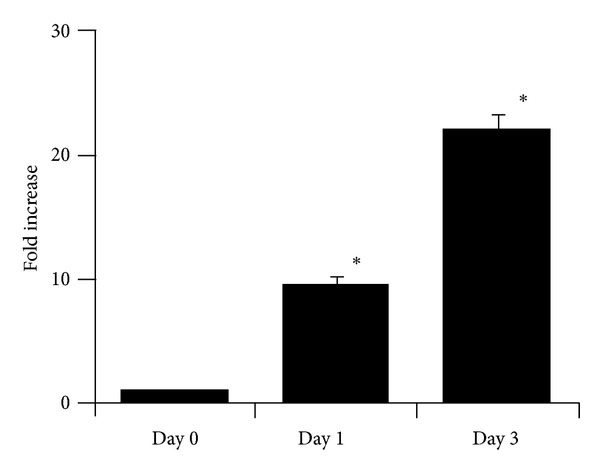
Fold increases of total cell numbers in peritoneal exudate during thioglycollate treatment *in vivo*. C57BL/6 mice (males) were treated with thioglycollate as described in Section 2. At three timepoints, before (day 0) and after (day 1 and 3) the treatment, peritoneal exudate cells were isolated and counted as described in Section 2. Fold increases were calculated as the cell number at a timepoint divided by the cell number on day 0 (defined as 1). Values are presented as the means ± SE. *n* = 3-4 per timepoint. *P < 0.001 versus day 0.

In our studies, we first determined the total cell numbers and numbers of neutrophils and MΦ in the peritoneal exudates before and after thioglycollate treatment. As shown in Figure 1, the total numbers of cells in the peritoneal exudates increased nearly 10-fold on day 1 (10.74 ± 0.54  × 10^6^ neutrophils and 5.48 ± 0.28  × 10^6^ MΦ; *n* = 3) compared to day 0 (0.87 ± 0.06  × 10^4^ neutrophils and 1.07 ± 0.07  × 10^6^ MΦ; *n* = 4), and reached peak levels on day 3 (1.94 ± 0.09  ×  10^6^ neutrophils and 40.19 ± 1.87  ×  10^6^ MΦ; *n* = 4). 

### 3.1. Inflammatory Mediator Concentrations in Peritoneal Exudates Reached the Peak on Day 1 Following Thioglycollate Treatment *In Vivo*

**Figure 2:**
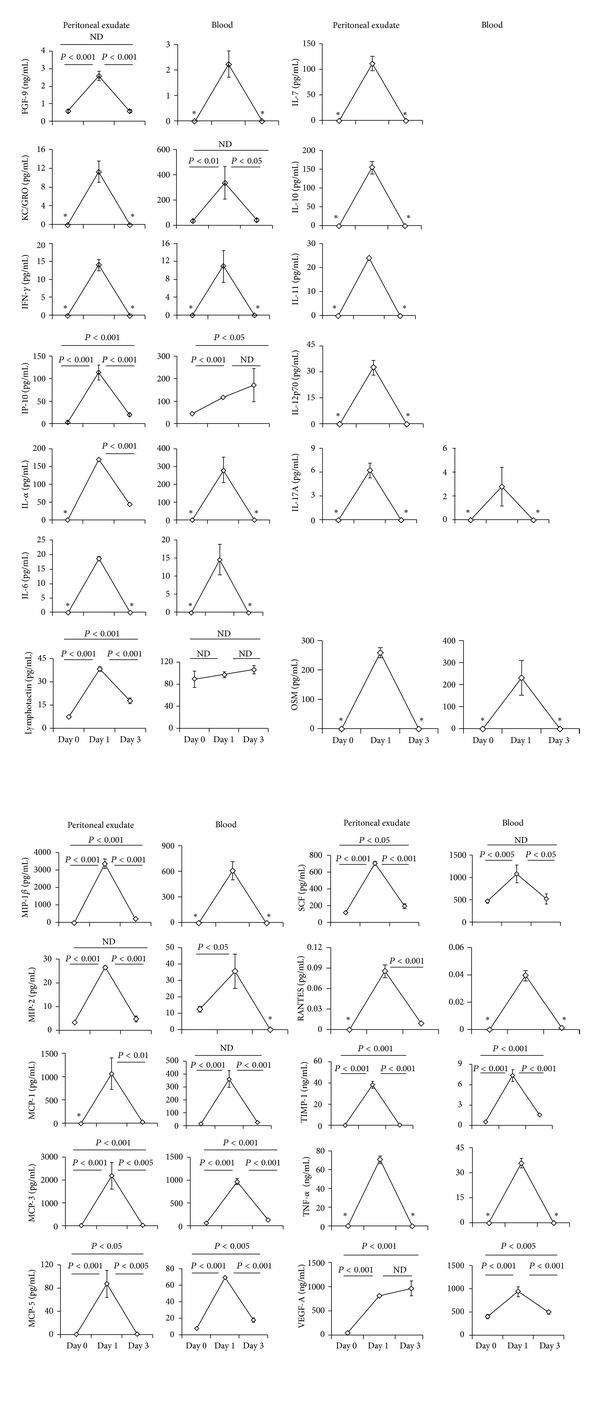
Concentration changes of 23 inflammatory mediators in peritoneal exudates and blood during thioglycollate treatment *in vivo*. C57BL/6 mice (males) were treated with thioglycollate as described in Section 2. At three timepoints, before (day 0) and after (day 1 and 3) the treatment, peritoneal exudates and plasma were isolated as described in Section 2. The concentrations of 23 inflammatory mediators were determined by a bead-based multiplexing immunoassay as described in Section 2. Values are represented as the means ± SE. *n* = 3–8 per timepoint. Data are from two independent experiments. *Denotes that the mediator was undetectable at the indicated timepoint.

We analyzed the concentrations of 23 inflammatory mediators at three timepoints in peritoneal exudates following thioglycollate treatment. As shown in Figure 2, the concentrations of all mediators reached peak levels on day 1. Based on the concentration levels in the exudates on day 1 (Table 1), these mediators were divided into three groups. The concentrations of the first group, which included MCP-1, MCP-3, FGF-9, MIP-1*β*, and TIMP-1, reached nanogram levels. Considering that these mediator concentrations were higher than those of other groups, these are likely the most important candidates to affect the induction of the MΦ accumulation on day 3. The concentrations of the mediators in the second group were determined to be in the picogram range, including IL-17A, KC/GRO, IFN-*γ*, IL-6, IL-11, MIP-2, IL-12p70, lymphotactin, TNF-*α*, MCP-5, IL-7, IP-10, IL-10, IL-1*α*, OSM, SCF, and VEGF-A. The concentrations of the third group were lower, in the femtogram range, including RANTES. Notably, in order to collect the peritoneal exudates, one milliliter of PBS was administered to the peritoneal cavities. Although we cannot accurately evaluate the volumes of original peritoneal fluid prior to PBS administration so far, our observation indicates that the volumes were lower than 0.5 mL. Thus, a 3-fold dilution before and after PBS administration was assumed in the evaluation of the original concentrations of the mediators in peritoneal exudates. 

### 3.2. Concentrations of Inflammatory Mediators *In Vivo* Were Generally Lower in Plasma than Those in Peritoneal Exudates on Day 1 after Thioglycollate Treatment

As shown in Figure 2 and similar to that observed in the exudates, the production of most mediators, except lymphotactin, reached peak levels in plasma on day 1. IL-7, IL-10, IL-11, and IL-12p70 were not detected in the plasma samples. Because comparisons of mediator concentrations between exudates and plasma would allow the source of the increased concentrations of mediators on day 1 to be identified, we calculated the ratios of mean values between exudates versus plasma for each mediator concentration. Based on our previous assumption that the exudates were likely diluted 3-fold, we chose 0.33 as an arbitrary threshold: if the ratio was higher than 0.33, the concentration of the mediator was likely higher in the exudates than in the plasma, and the source of the increased concentrations of the mediators was likely to be in the exudates. If the ratio was lower than 0.33, the concentration of the mediator was likely higher in the plasma than in the exudates, and the source of the increased concentrations of the mediators was likely to be in the plasma. There were 19 mediators with detectable concentration levels in both exudates and plasma (Table 1). Among these, 5 mediators exhibited ratios higher than 0.33 in addition to significant differences between the exudate and plasma concentrations (*P* < 0.05); these included lymphotactin, MIP-1*β*, RANTES, TIMP-1, and TNF-*α*, indicating their origins from exudates. Thirteen mediators exhibited ratios higher than 0.33 and had no significant differences between exudate and plasma concentrations (*P* > 0.05); these included MCP-1, MCP-3, FGF-9, IL-6, MIP-2, IFN-*γ*, MCP-5, IP-10, IL-1*α*, IL-17A, OSM, SCF, and VEGF-A. However, a clear trend was observed in which the concentrations in exudates were higher than those in plasma, indicating that these mediators were highly likely to originate in the exudates. Only one mediator, KC/GRO, had a ratio lower than 0.33 and an insignificant difference between exudate and plasma concentrations (*P* > 0.05). These findings indicate that, for most of inflammatory mediators, the increases in mediator concentrations on day 1 likely originate from the exudates. 

### 3.3. Inflammatory Mediator Concentrations *In Vivo* Were Generally Significantly Reduced in Peritoneal Exudates on Day 3 versus Day 1 after Thioglycollate Treatment

As shown in Figure 2, there were ten mediators for which the concentrations in the exudates were below the detection limits at both day 0 and day 3; these included KC/GRO, IL-17A, IFN-*γ*, IL-6, IL-11, IL-12p70, TNF-*α*, IL-7, IL-10, and OSM. There were 3 mediators, RANTES, MCP-1, and IL-1*α*, for which the concentrations in exudates were detectable on day 3, but not day 0; the concentrations of these mediators were significantly reduced on day 3 when compared to those on day 1 (*P* < 0.01). Another group of 10 mediators were detected at all three timepoints; except for VEGF-A, the concentrations of these mediators were significantly reduced on day 3 compared to day 1 (*P* < 0.005). Among these 10 mediators, the day 3 concentrations of FGF-9 and MIP-2 reduced to the levels of those on day 0 (*P* > 0.05). Thus, the concentrations of the mediators (except VEGF-A) in peritoneal exudates on day 3 were significantly reduced compared to those on day 1. 

### 3.4. Basal Concentrations of Inflammatory Mediators *In Vivo* Are Higher in Plasma than in Exudates

As shown in Figure 2, there were eight inflammatory mediators that were detected in both plasma and exudates on day 0: IP-10, lymphotactin, MIP-2, MCP-3, MCP-5, SCF, TIMP-1, and VEGF-A.  Table 2 compared the concentrations of these detectable mediators and calculated the ratios of mean values between plasma and exudate concentrations. Using an estimated threshold of 0.33 as previously described, the concentrations of these eight mediators were significantly higher in plasma than in exudates at basal conditions (*P* < 0.001). 

### 3.5. LPS Induces the Production of Inflammatory Mediators *In Vitro* in Peritoneal MΦ That Were Isolated from Mice Treated with Thioglycollate for Three Days

In contrast to the maximum numbers of peritoneal MΦ that were observed on day 3 after thioglycollate treatment (Figure 1), the general production of inflammatory mediators was reduced significantly at this timepoint in both peritoneal exudates and blood (Figure 2). Thus, an intriguing question is whether the peritoneal MΦ switch to a subset with an irreversibly low capacity to produce these mediators between days 1 and 3. To address this issue, peritoneal MΦ were isolated on day 3, purified, and treated with LPS for 24 hours. As shown in Table 3, after the treatment, inflammatory mediator productions were markedly increased in both cell culture media and lysates. There were eleven mediators with greater than 10-fold production increases in culture media; these included IL-1*α*, MIP-2, MCP-1, MCP-3, IFN-*γ*, TIMP-1, TNF-*α*, IP-10, MCP-5, IL-6, and MIP-1*β*. Notably, the fold increases in mediator concentrations between culture media and cell lysates were generally consistent (*P* > 0.05), except for IL-6, MCP-3, RANTES, and TIMP-1, hinting the participation of posttranslational mechanisms in the regulation of the production of these mediators. 

## 4. Discussion

In this study, we investigated the spatiotemporal profiling of multiple inflammatory mediators at different times (days 0, 1, and 3) and in different locations (peritoneal cavity and blood vessels) during thioglycollate treatment *in vivo*. Thus, our discussion will focus on two immune properties that were revealed by the investigation of multiple mediators, rather than the biological significance of a specific mediator. 

### 4.1. Chemotactic Gradient between Blood and Peritoneal Exudates

Leukocyte accumulation in inflamed tissues is a vital immune response to inflammation, which is guided by chemotactic gradients of a goup of proinflammatory mediators. Indeed, thioglycollate induced peritonitis has been widely used as a model with which to investigate the mechanisms behind the correlation between chemotactic gradients and leukocyte accumulation. Using this model, proteins that are newly identified in the process of leukocyte accumulation include a heterotrimeric G_i_-protein G_*α*i2_  [16], polypeptide N-acetylgalactosamine transferase-1 [17], SLP-76 [18], ADAP [18], and sphingosine 1-phosphate receptor 2 [19]. Our current studies provided additional evidence to demonstrate these concepts, in which at basal levels, the mediator concentrations generally in the blood were higher than those in exudates (Table 2). This finding indicates the presence of a reversed chemotactic gradient at basal levels between the blood vessels and peritoneal cavity, which likely plays a critical role in the prevention of MΦ emigration from circulating peripheral blood. Although only some of the mediators show significant differences between exudates and blood on day 1 following thioglycollate treatment, a trend is obvious (Table 1): the mean value ratios of all detectable mediator concentrations in the exudates versus blood were below 0.33 (down to 0.07) on day 0, while all ratios were above 0.33 (up to 9.75) on day 1. This finding indicates that although inflammatory mediator concentrations in both exudates and blood were increased one day after thioglycollate treatment, the increased concentrations of most mediators in exudates were higher than in blood. These results elucidate that overlapping chemoattractant gradients exist between the blood and peritoneal exudates on day 1 after thioglycollate treatment, which allows MΦ migration to reach maximum levels on day 3. 

Several models have been proposed previously to evaluate chemotactic gradients, such as the *in vitro* agarose gel-filled tissue culture dish [20] and *ex vivo* cocultures with mouse cremaster muscle in agarose gel [21]. Our studies provide evidence that thioglycollate induced peritonitis is another model with which to study chemotactic gradients *in vivo*, such as genetically modified mice. 

### 4.2. Inhibition of Inflammatory Mediator Production in MΦ

We were surprised to discover initially that the productions of nearly all inflammatory mediators in both exudates and blood were reduced on day 3 following thioglycollate treatment, because MΦ are believed to be major producers for inflammatory mediators and MΦ numbers reach peak levels on day 3. However, this result can be interpreted by the previous finding that the phagocytosis of apoptotic neutrophils by MΦ results in the inhibition of proinflammatory mediator production [22]. Although this previous finding is based on *in vitro* models, our result hints that this early finding could be applied to *in vivo* models. Because increased cell death, as characterized by early and late apoptotic cells, can be detected as early as 4 hours after thioglycollate treatment [23], it is highly likely that MΦ gradually lose the ability to produce inflammatory mediators after taking up apoptotic cells *in vivo*. However, there are some differences between our *in vivo* study and the previous *in vitro* study. For example, previous studies showed that treatment with apoptotic cells during monocyte activation increased the production of anti-inflammatory cytokine IL-10, whereas in our model, IL-10 production was not increased on day 3, hinting that regulation of IL-10 production *in vivo* is more complicated. 

Our studies also showed that the increased neutrophil and MΦ accumulation promote inflammatory mediator productions within the first day after thioglycollate treatment, indicating that the level of neutrophil and MΦ accumulation determines the intensity of inflammation in the early stage of inflammation. The resolution stage of inflammation is defined as the interval from maximum neutrophilic accumulation to the point at which neutrophils are fully removed from the tissue [24]. During this stage, neutrophils are undergoing apoptosis and are subsequently cleared by MΦ and other phagocytic cells. Our studies also indicated that in the resolution stage of inflammation, increased MΦ accumulation is not associated with the mediator productions and the level of MΦ accumulation likely determines the persistence of inflammation. 

Extensive evidence from both clinical studies and animal models has implicated the dysregulation of inflammatory mediators as a contributing factor in the pathophysiological progression of cardiovascular disease, such as atherosclerosis [25], abdominal aortic aneurysm, and cardiac infarction [26]. In addition, peritoneal MΦ are a reasonable model for mimicking cardiovascular MΦ responses [27, 28], including foam cell formation [29, 30], MΦ recruitment [31, 32], MΦ apoptosis [33], and cytokine production [34]. In this regard, the regulation of MΦ accumulation has been proposed as a therapeutic approach to regulate inflammatory mediator production [35, 36]. Our results hint that the optimal period of this therapeutic intervention is likely to be in the early stage of inflammation. 

## Figures and Tables

**Table 1:** Concentrations and ratios of inflammatory mediators in exudates and plasma on day 1 after thioglycollate treatment in vivo *.

	Exudates (A, *n* = 3)	Plasma (B, *n* = 4)	Ratio of mean value between A and B
FGF-9	2.60 ± 0.26	2.23 ± 0.51	1.17
KC/GRO	11.33 ± 2.33	335.00 ± 127.05	0.03
IFN-*γ*	14.00 ± 1.53	10.90 ± 3.52	1.28
IP-10	114.00 ± 16.70	117.50 ± 5.72	0.97
IL-1*α*	170.33 ± 3.53	282.00 ± 71.17	0.60
IL-6	18.67 ± 0.67	14.65 ± 4.24	1.27
IL-17A	6.23 ± 0.92	2.81 ± 1.62	2.22
Lymphotactin**	38.70 ± 1.45	98.00 ± 5.28	0.40
MIP-1β**	3373.33 ± 274.73	610.25 ± 105.45	5.53
MIP-2	26.70 ± 0.67	35.75 ± 10.38	9.75
MCP-1	1069.33 ± 341.10	364.25 ± 65.35	2.94
MCP-3	2193.33 ± 579.55	970.50 ± 64.64	2.26
MCP-5	89.00 ± 21.50	69.25 ± 1.65	1.29
OSM	260.00 ± 17.32	231.25 ± 79.01	1.12
SCF	707.00 ± 21.55	1084.50 ± 198.52	0.65
RANTES***	0.09 ± 0.01	0.04 ± 0.00	2.17
TIMP-1**	38.67 ± 3.18	7.40 ± 0.85	5.23
TNF-α**	71.67 ± 2.96	35.75 ± 2.93	2.01
VEGF-A	820.33 ± 18.44	943.00 ± 109.18	0.87

*The unit for all concentrations is pg/mL, except FGF-9 and TIMP-1 as ng/mL.

**P < 0.001.

***P < 0.05.

All other samples *P* > 0.05.

**Table 2:** Concentrations of inflammatory mediators in exudates and plasma at basal condition *in vivo. *

	Exudates (A, *n* = 6)	Plasma (B, *n* = 8)	Ratio of mean value between A and B on day 0	Ratio on day 1
IP-10	3.05 ± 0.12	44.00 ± 4.88	0.07	0.97
lymphotactin	7.57 ± 0.68	89.50 ± 15.23	0.09	0.40
MIP-2	3.63 ± 0.26	12.59 ± 1.61	0.29	9.75
MCP-3	3.42 ± 0.16	59.50 ± 2.66	0.06	2.26
MCP-5	0.73 ± 0.10	7.83 ± 1.32	0.09	1.29
SCF	119.00 ± 6.88	472.00 ± 39.11	0.25	0.65
TIMP-1	0.20 ± 0.02	0.61 ± 0.05	0.32	5.23
VEGF-A	50.62 ± 12.06	403.50 ± 43.57	0.13	0.87

(1) *P* < 0.001 between A and B for all groups.

(2) The unit for all concentrations is pg/mL, except TIMP-1 as ng/mL.

(3) The ratio on day 1 is derived from Table 1.

**Table 3:** Fold increases of inflammatory mediator productions in cell culture media and lysates of murine peritoneal macrophages following LPS treatment for 24 h *in vitro. *

	Fold increase	
	Culture media (A, *n* = 2)	Cell lysates (B, *n* = 4)
IL-11	1.66 ± 0.03	4.65 ± 1.30
OSM	1.78 ± 0.10	11.91 ± 3.55
IL-7	1.90 ± 0.16	8.75 ± 2.82
SCF	1.94 ± 0.15	11.86 ± 3.30
Lymphotactin	1.98 ± 0.22	6.14 ± 1.61
FGF-9	2.22 ± 0.23	2.48 ± 1.10
IL-10	3.29 ± 0.08	7.71 ± 2.33
IL-12p70	4.36 ± 0.93	2.20 ± 0.96
VEGF-A	4.81 ± 1.36	2.72 ± 0.53
KC/GRO	6.21 ± 2.87	17.28 ± 5.00
RANTES**	8.93 ± 3.00	1.87 ± 0.55
IL-1*α*	10.44 ± 2.36	225.12 ± 126.41
MIP-2	12.61 ± 6.43	25.01 ± 11.05
MCP-1	14.17 ± 0.80	12.95 ± 1.56
MCP-3**	17.69 ± 1.20	11.44 ± 0.98
IFN-*γ*	17.94 ± 1.23	29.28 ± 11.75
TIMP-1**	20.87 ± 3.31	10.32 ± 0.97
TNF-*α*	20.93 ± 8.58	42.44 ± 17.07
IP-10	44.65 ± 14.11	28.17 ± 7.03
MCP-5	47.69 ± 23.83	3.72 ± 0.32
IL-6*	81.43 ± 11.59	33.04 ± 4.43
MIP-1*β*	127.89 ± 103.35	20.52 ± 9.87

*P < 0.01 A versus B.

**P < 0.05 A versus B.

All other groups, *P* > 0.05.

## Abbreviations

FBS: Fetal bovine serum
FGF: Fibroblast growth factor
IFN: Interferon
IL: Interleukin
i.p.: Intraperitoneal
IP-10: IFN-*γ*-inducible protein 10
KC/GRO: Growth-regulated *α* protein
LDD: Least detectable dose
LPS: Lipopolysaccharide
MCP: Monocyte chemotactic protein
MIP: Macrophage inflammatory protein
MΦ: Macrophages
OSM: Oncostatin-M
RANTES: T-cell-specific protein
SCF: Stem cell factor
SE: Standard error
TIMP: Tissue inhibitor of metalloproteinases
TNF: Tumor necrosis factor
VEGF: Vascular endothelial growth factor.

## References

[1] McGrath EE, Marriott HM, Lawrie A, Francis SE, Sabroe I (2011). TNF-related apoptosis-inducing ligand (TRAIL) regulates inflammatory neutrophil apoptosis and enhances resolution of inflammation. *Journal of Leukocyte Biology*.

[2] Kim HW, Chan Q, Afton SE (2012). Human macrophage ATP7A is localized in the *trans*-Golgi apparatus, controls intracellular copper levels, and mediates macrophage responses to dermal wounds. *Inflammation*.

[3] Broche F, Tellado JM (2001). Defense mechanisms of the peritoneal cavity. *Current Opinion in Critical Care*.

[4] Williams JC, Wagner NJ, Earp HS, Vilen BJ, Matsushima GK (2010). Increased hematopoietic cells in the mertk-/- mouse peritoneal cavity: a result of augmented migration. *Journal of Immunology*.

[5] Moro K, Yamada T, Tanabe M (2010). Innate production of TH 2 cytokines by adipose tissue-associated c-Kit+ Sca-1+ lymphoid cells. *Nature*.

[6] Topley N, Brown Z, Jorres A (1993). Human peritoneal mesothelial cells synthesize interleukin-8: synergistic induction by interleukin-1*β* and tumor necrosis factor-*α*. *American Journal of Pathology*.

[7] Topley N, Liberek T, Davenport A, Li FK, Fear H, Williams JD (1996). Activation of inflammation and leukocyte recruitment into the peritoneal cavity. *Kidney International*.

[8] Ajuebor MN, Das AM, Virág L, Flower RJ, Szabó C, Perretti M (1999). Role of resident peritoneal macrophages and mast cells in chemokine production and neutrophil migration in acute inflammation: evidence for an inhibitory loop involving endogenous IL-10. *Journal of Immunology*.

[9] Melnicoff MJ, Horan PK, Morahan PS (1989). Kinetics of changes in peritoneal cell populations following acute inflammation. *Cellular Immunology*.

[10] Bellingan GJ, Caldwell H, Howie SEM, Dransfield I, Haslett C (1996). *In vivo* fate of the inflammatory macrophage during the resolution
of inflammation: inflammatory macrophages do not die locally, but
emigrate to the draining lymph nodes. *Journal of Immunology*.

[11] Casrouge A, Decalf J, Ahloulay M (2011). Evidence for an antagonist form of the chemokine CXCL10 in patients chronically infected with HCV. *Journal of Clinical Investigation*.

[12] Joseph SB, Bradley MN, Castrillo A (2004). LXR-dependent gene expression is important for macrophage survival and the innate immune response. *Cell*.

[13] Zavitz CCJ, Bauer CMT, Gaschler GJ (2010). Dysregulated macrophage-inflammatory protein-2 expression drives illness in bacterial superinfection of influenza. *Journal of Immunology*.

[14] Blumberg H, Dinh H, Trueblood ES (2007). Opposing activities of two novel members of the IL-1 ligand family regulate skin inflammation. *Journal of Experimental Medicine*.

[15] Camargo JF, Quinones MP, Mummidi S (2009). CCR5 expression levels influence NFAT translocation, IL-2 production, and subsequent signaling events during T lymphocyte activation. *Journal of Immunology*.

[16] Wiege K, Le DD, Syed SN, Ali SR, Novakovic A (2012). Defective macrophage migration in G*α*i2- but not G*α*i3-deficient mice. *The Journal of Immunology*.

[17] Block H, Ley K, Zarbock A (2012). Severe impairment of leukocyte recruitment in ppGalNAcT-1-deficient mice. *The Journal of Immunology*.

[18] Block H, Herter JM, Rossaint J, Stadtmann A, Kliche S (2012). Crucial role of SLP-76 and ADAP for neutrophil recruitment in mouse kidney ischemia-reperfusion injury. *The Journal of Experimental Medicine*.

[19] Michaud J, Im DS, Hla T (2010). Inhibitory role of sphingosine 1-phosphate receptor 2 in macrophage recruitment during inflammation. *Journal of Immunology*.

[20] Foxman EF, Campbell JJ, Butcher EC (1997). Multistep navigation and the combinatorial control of leukocyte chemotaxis. *Journal of Cell Biology*.

[21] Cara DC, Kaur J, Forster M, McCafferty DM, Kubes P (2001). Role of p38 mitogen-activated protein kinase in chemokine-induced emigration and chemotaxis *in vivo*. *Journal of Immunology*.

[22] Fadok VA, Bratton DL, Konowal A, Freed PW, Westcott JY, Henson PM (1998). Macrophages that have ingested apoptotic cells *in vitro* inhibit proinflammatory cytokine production through autocrine/paracrine mechanisms involving TGF-*β*, PGE2, and PAF. *Journal of Clinical Investigation*.

[23] Wan H, Coppens JMC, Van Helden-Meeuwsen CG (2009). Chorionic gonadotropin alleviates thioglycollate-induced peritonitis by affecting macrophage function. *Journal of Leukocyte Biology*.

[24] Serhan CN, Brain SD, Buckley CD (2007). Resolution of inflammation: state of the art, definitions and terms. *FASEB Journal*.

[25] Little PJ, Chait A, Bobik A (2011). Cellular and cytokine-based inflammatory processes as novel therapeutic targets for the prevention and treatment of atherosclerosis. *Pharmacology and Therapeutics*.

[26] Srinivas G, Anversa P, Frishman WH (2009). Cytokines and myocardial regeneration: a novel treatment option for acute myocardial infarction. *Cardiology in Review*.

[27] Li X, Mikhalkova D, Gao E (2011). Myocardial injury after ischemia-reperfusion in mice deficient in Akt2 is associated with increased cardiac macrophage density. *American Journal of Physiology, Heart and Circulatory Physiology*.

[28] Tsujita K, Kaikita K, Hayasaki T (2007). Targeted deletion of class A macrophage scavenger receptor increases the risk of cardiac rupture after experimental myocardial infarction. *Circulation*.

[29] Levin MC, Jirholt P, Wramstedt A, Johansson ME, Lundberg AM (2011). Rip2 deficiency leads to increased atherosclerosis despite decreased inflammation. *Circulation Research*.

[30] Yakubenko VP, Bhattacharjee A, Pluskota E, Cathcart MK (2011). *α*m*β*2 integrin activation prevents alternative activation of human and murine macrophages and impedes foam cell formation. *Circulation Research*.

[31] Keul P, Lucke S, Von Wnuck Lipinski K (2011). Sphingosine-1-Phosphate receptor 3 promotes recruitment of monocyte/macrophages in inflammation and atherosclerosis. *Circulation Research*.

[32] Pagler TA, Wang M, Mondal M (2011). Deletion of ABCA1 and ABCG1 impairs macrophage migration because of increased Rac1 signaling. *Circulation Research*.

[33] Yvan-Charvet L, Pagler TA, Seimon TA (2010). ABCA1 and ABCG1 protect against oxidative stress-induced macrophage apoptosis during efferocytosis. *Circulation Research*.

[34] Wang X, Jin W, Rader DJ (2007). Upregulation of macrophage endothelial lipase by toll-like receptors 4 and 3 modulates macrophage interleukin-10 and -12 production. *Circulation Research*.

[35] Mackay CR (2008). Moving targets: cell migration inhibitors as new anti-inflammatory therapies. *Nature Immunology*.

[36] Friedl P, Weigelin B (2008). Interstitial leukocyte migration and immune function. *Nature Immunology*.

